# The dynamic collapse of the trachea during anesthesia for a pediatric patient with a large anterior mediastinal mass: A case report

**DOI:** 10.1002/ccr3.3005

**Published:** 2020-06-11

**Authors:** Naoko Niimi, Kumi Kataoka, Masakazu Hayashida, Eiichi Inada

**Affiliations:** ^1^ Department of Anesthesia Juntendo University Hospital Bunkyo‐ku Japan

**Keywords:** airway collapse, airway management, large anterior mediastinal mass, pediatric anesthesia

## Abstract

Anesthesia for patient with large anterior mediastinal mass might induce life‐threatening complication. Maintaining the spontaneous breathing throughout the procedure and finding rescue position are the cornerstones of anesthetic management.

## Graphical abstract

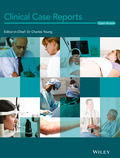

## 1 INTRODUCTION

Anesthesia for patients with large anterior mediastinal masses is challenging because of dynamic life‐threatening airway obstruction and cardiovascular compromise by the mass.^1^, ^2^ We report a pediatric patient with a rapidly enlarging anterior mediastinal mass who successfully underwent emergency tumor biopsy under general anesthesia. Publishing this case report is approved by our ethics committee, and parents gave consent to publish the details of the patient's case.

## 2 CASE PRESENTATION

A 13‐year‐old previously healthy boy was admitted to our hospital for cough and orthopnea. Chest X‐ray showed a remarkably enlarged mediastinum, and chest CT revealed a large anterior mediastinal mass measuring 113 × 114 × 69 mm compressing the mid‐distal trachea, bilateral bronchi (left > right), and the superior vena cava. There was no compression of the upper trachea or the pulmonary artery.

As there were no palpable superficial lymph nodes to resect for biopsy, we scheduled an emergency thoracotomy for tumor biopsy in the right recumbency.

The patient had only a mild cough and no dyspnea in the sitting position in the ward. However, he could not lie supine for the past few days because his cough and dyspnea had worsened.

The patient arrived in the operation room in the sitting position receiving oxygen at 2 L/min by nasal cannula. The patient's peripheral capillary oxygen saturation (SpO2) was 94%, and the arterial partial pressure of oxygen (PaO2) was 61.8 Torr. During the epidural catheter insertion in the right lateral recumbency, the patient's SpO2 increased remarkably to 100% (PaO2: 208.2 Torr). We decided to induce general anesthesia with the patient in the right lateral recumbency to maintain better oxygenation. After two boluses of ketamine at 0.5 mg/kg, 5 minutes apart, we administered remifentanil infusion at 0.05 mcg/kg/min, dexmedetomidine at 0.4 mcg/kg/h, and propofol at 200 mcg/kg/min, and topically anesthetized the patient's larynx, pharynx, epiglottis, and trachea with 4% lidocaine spray. Fiberoptic tracheal intubation with a Taper Guard^®^™ (Medtronic) 6‐mm tracheal tube and an Ambu^®^ aScope™ 4 Broncho Slim 3.8/1.2 (Ambu Corp) was performed smoothly under conscious sedation. After intubation, we maintained the same doses of the infusions and gave an epidural bolus of 9 mL of 0.25% ropivacaine. Thoracotomy was then performed through the left fifth intercostal space. We did not administer muscle relaxants throughout the procedure.

Following the biopsy, we changed the patient's position to supine to insert a central venous catheter for subsequent chemotherapy. He was breathing regularly and effectively at first, and dynamic changes in the tracheal opening were observed (Video S1).

Approximately 30 minutes after changing the position, the patient started to cough, and the capnogram tracing flattened. We were not able to manually ventilate the patient using positive pressure, and his SpO2 level decreased to 70%, and his blood pressure decreased to 70/20 mm Hg. After immediately repositioning the patient to right lateral recumbency, both SpO2 and blood pressure returned to baseline values, and the capnogram tracing was again visible. We abandoned the central venous catheter insertion because of the risk of catastrophic airway compromise.

The diagnosis of the tumor was T‐cell lymphoblastic lymphoma, and the patient underwent 9 months of chemotherapy, achieving complete remission.

## 3 DISCUSSION

Tracheal collapse secondary to positioning the patient could have been catastrophic. Anghelescu et al^3^ identified orthopnea, upper body edema, compression of the great vessels, and compression of the trachea/main stem bronchus as risk factors for anesthesia in children with anterior mediastinal masses. Considering that the patient had three risk factors, we recommended chemotherapy first, to reduce the tumor volume before biopsy and prevent fatal cardiorespiratory collapse. However, after a discussion with the pediatrician, we decided to obtain a pathological diagnosis first, to have an accurate diagnosis and provide effective treatment.

Our options for anesthesia for thoracotomy for this patient were epidural anesthesia or combined general/epidural anesthesia. Epidural anesthesia with or without light sedation might lower the risk of airway collapse. However, in an emergency, further immediate treatment might be difficult, and pediatric patients may not tolerate operation room situations or invasive procedures, depending on their age and personality traits. General anesthesia is less stressful for patients and is easier when they are not cooperative. Additionally, once the airway is secured with tracheal intubation, maintenance during the procedure might be easier. However, during general anesthesia induction, one of the biggest challenges is to secure the airway and prevent cardiovascular and respiratory collapse because anesthetics, especially muscle relaxants, decrease tone in the body, and can change physiological states. Considering all of these factors, we chose combined general and epidural anesthesia with tracheal intubation, and we decided to avoid muscle relaxants, to maintain spontaneous breathing.

Another consideration was choice of the tracheal tube. The tumor was compressing bilateral main bronchi, and the internal diameters were left: 2 mm and right: 6 mm according to chest CT. Tubes with such small diameters and sufficient lengths to pass through the right bronchus were not available; therefore, bronchial intubation was not possible. Our decision was a Taper Guard^®^™ (Medtronic) 6‐mm single‐lumen tracheal tube to secure at least the upper airway and tracheal airway.

## 4 CONCLUSION

By finding a rescue position and maintaining spontaneous breathing, we successfully performed anesthesia for emergent thoracotomy biopsy in a 13‐year‐old boy with a large anterior mediastinal mass.

## CONFLICT OF INTEREST

The authors have no conflict of interest to declare about this article.

## AUTHOR CONTRIBUTION

NN and KK: participated in the management of this patient as well as in the preparation and edition of this manuscript. MH and EI: involved in manuscript writing.

## Supporting information

Video S1

Video S1

## Data Availability

Data openly available in a public repository that issues datasets with DOIs.
