# Supplementary material for: The dynamic collapse of the trachea during anesthesia for a pediatric patient with a large anterior mediastinal mass: A case report
Source: Clin Case Rep. 2020 Jun 11;8(9):1814–5. doi: 10.1002/ccr3.3005 (PMC7495815; doi:10.1002/ccr3.3005)
Supplement: Supplementary file 2 — Video S1 [file CCR3-8-1814-s002.docx]

**Video S1**. The dynamic change in bronchoscopy of the trachea and bronchi (left > right) in a supine position
